# Extraction and Analysis of Phenolic Compounds from Rocket: Development of a Green and Innovative DES-Based Extraction Method

**DOI:** 10.3390/molecules30051177

**Published:** 2025-03-06

**Authors:** Vittoria Terrigno, Susanna Della Posta, Giorgia Pietrangeli, Teodora Chiara Tonto, Vittoria Locato, Laura De Gara, Chiara Fanali

**Affiliations:** Department of Science and Technology for Sustainable Development and One Health, Università Campus Bio-Medico di Roma, Via Álvaro del Portillo 21, 00128 Rome, Italy; vittoria.terrigno@unicampus.it (V.T.); giorgia.pietrangeli@unicampus.it (G.P.); t.tonto@unicampus.it (T.C.T.); v.locato@unicampus.it (V.L.); l.degara@unicampus.it (L.D.G.); c.fanali@unicampus.it (C.F.)

**Keywords:** deep eutectic solvent, *Eruca sativa*, HPLC-MS, phenolic compounds, green chemistry

## Abstract

*Eruca sativa* Mill. is an annual plant belonging to the Cruciferous family that is characterized by the presence of antioxidant bioactive molecules such as phenolic compounds. Their extraction is usually performed through solid–liquid extraction based on the use of organic solvent. Deep eutectic solvents (DESs) are new green solvents capable of increasing bioactive molecules yield if replaced with organic solvents. The aim of this work was to develop a green analytical method based on the use of DESs for the determination of phenolic compounds in rocket plants. The extraction optimization involved the selection of the best extraction solvent among different selected DESs and the study of the parameters that mainly affect the extraction yield: the quantity of water to add to the selected DES to reduce its viscosity, the matrix-to-solvent ratio, and the time and temperature of the extraction. ChCl-glucose (1:2 molar ratio) DES was selected as the extraction solvent under the following optimized conditions: 1:50 (*w*/*v*) as the matrix-to-solvent ratio; 30% of water was added to the DES; extraction time of 30 min; and extraction temperature of 50 °C. The rocket phenolic compounds profile was determined through a high-performance liquid chromatography coupled with mass spectrometry (HPLC-MS) analysis. The innovative green method was applied to real plant samples to determine the growth conditions that favored the accumulation of bioactive molecules.

## 1. Introduction

### 1.1. Eruca sativa Mill: Phenolic Compounds Analysis

Rocket (*Eruca sativa* Mill.) is a green leafy vegetable belonging to the *Brassicaceae* family [1]. It is an edible annual herbaceous plant native to the area of the Mediterranean Basin and Central-Western Asia. The term “rocket” refers mainly to the *Eruca* and *Diplotaxis* genera within the *Cruciferae* family [2]. Nowadays, it is widely cultivated in other parts of the world [3] (USA, UK, Australia, Israel) and extremely utilized in the alimentary field [4]. For its growth, it needs a small quantity of water and reaches maturity very quickly. It can also grow spontaneously, and in this case, the taste is more decided and the leaf is harder and darker. In recent years, rocket species have been extensively studied for their powerful antioxidant properties [1]. In fact, these vegetables are rich in health-promoting phytochemicals such as fibers, carotenoids, glucosinolates, and phenolic compounds with positive effects on human health [1]. To date, it is believed that regular consumption of food antioxidants can reduce the risk of several diseases such as inflammatory diseases and cancer risk [5]. Phenolic compounds are secondary metabolites structurally characterized by the presence of aromatic groups with one or more hydroxyl groups. These bioactive molecules have a high antioxidant capacity, which positively affects human health. The most abundant phenolic compounds found in rocket (*Eruca sativa* Mill.) belong to the family of hydroxycinnamic acids (ferulic acid, sinapic acid-glucose, 3-caffeoylquinic acid) and flavonoids (quercitin; quercitin-3-O-galactoside; kaempferol-3-O-glucuronide; isorhamnetin-3-O-rutinoside; apigenin 7-O-glucoside) [6,7]. However, the phenolic profile is strongly variable, and it depends on several factors such as the species of the rocket, geographical area of origin, climate, and sunlight. As reported by Matev et al. [8], the geographical area of origin of the plants of *Eruca sativa* Mill. influence the composition of bioactive compounds and the total phenolic content (TPC) of rocket; in fact, in the study conducted by Matev et al. [8], it was observed that plants with Bulgarian origins possessed a higher TPC than plants with Italian origins (0.9 and 4.7 mg/g fresh weight) [8]. The main factors of cultivation in greenhouses that modulate the concentration of polyphenols are the UV radiation of plants and growth temperature [9]. Data reported in literature state that different parts of *Eruca sativa* Mill. (flowers, seeds, leaves, stem) contain a different quantity of phenolic compounds, showing how the seeds contained a greater TPC than the aerial parts of the plant [10].

Phenolic compounds analysis in *Eruca sativa* involved an extraction step with a specific organic extraction solvent and subsequent qualitative and quantitative analysis through a spectrophotometric assay or chromatographic techniques coupled with mass spectrometry [6,8]. Concerning the sample preparation step, the extraction of phenolic compounds from different rocket parts of the plant could be carried out by supercritical CO_2_ (SCO_2_) and mostly by solid–liquid extraction (SLE), using methanol, ethanol, or acidified ethanol as the main extraction solvent, performing a liquid nitrogen treatment before the extraction [11,12,13]. With the aim of pursuing and respecting the requirements of “*green chemistry*” [14], research is increasingly aiming to develop a miniaturized extraction procedure, based on the use of low volumes and quantities of extraction solvent and sample, respectively, and replace traditional organic solvents with innovative solvents in a green perspective [15].

### 1.2. Green Chemistry in Sample Preparation: Deep Eutectic Solvent

Deep eutectic solvents (DESs) are new-generation solvents, characterized by different properties in accordance with green chemistry principles. DESs are an emerging class of solvents composed of two or more components that interact through hydrogen bonding, forming a eutectic mixture with a melting point significantly lower than that of the individual components. Major hydrogen bond acceptors (HBAs) include choline chloride (ChCl), betaine, and acetylcholine, while a wide range of compounds act as hydrogen bond donors (HBDs), such as organic acids, sugars, amines, and polyalcohols. Due to their high biodegradability, low toxicity, and ease of preparation, DESs are considered a green and sustainable alternative to conventional solvents. Additionally, they are cost-effective, making them economically viable for various industrial applications. A special subclass of these solvents, known as natural deep eutectic solvents (NADESs), consists exclusively of naturally derived components, further enhancing their biocompatibility and expanding their potential use in pharmaceuticals, cosmetics, food processing, and biotechnology [16,17]. From the data in the literature, there is no evidence of the use of DESs for the extraction of phenolic compounds from *Eruca sativa* Mill. However, there are works such as that of Pavić et al. [18], in which an NADES composed of ChCl and citric acid is used as an extractive solvent for phenolic compounds from the plant species *Ruta graveolens* L. Cao et al. [19] performed a green extraction of six phenolic compounds from Rattan (*Calamoideae faberii*) using ChCl-ethylene glycol as an extractive solvent. One of the key challenges in utilizing deep eutectic solvents (DESs) for extraction processes is their inherently high viscosity, which can significantly limit mass transfer and diffusion efficiency. A widely used strategy to overcome this drawback is the addition of a controlled amount of water, which effectively lowers viscosity while preserving the solvent’s essential properties. However, it is crucial to carefully regulate the water content, as excessive dilution can disrupt the hydrogen bonding network, potentially reducing the solvent’s selectivity and extraction capability. Moreover, the addition of water can also influence other physicochemical properties of DESs, such as polarity and solubility, which must be considered when optimizing their application [20]. The aim of the work was to develop a green analytical method based on the use of DESs for the determination of phenolic compounds in rocket plants. The method optimization first involved the selection, among a series of tested DESs, of the one that was guaranteed the best phenolic compounds extraction yield. Then, a percentage of water to add to the DES, and the matrix-to-solvent ratio and time and temperature of the extraction were studied. The optimized method was then applied for the analysis of rocket samples grown in different conditions, such as temperature and salt stress induced by sodium chloride (NaCl) (0 mM, 150 mM and 300 mM of NaCl), in order to select the growth conditions that favored the accumulation of phenolic compounds in the plant. The phenolic compounds profile was determined using high-performance liquid chromatography coupled with mass spectrometry (HPLC-MS) analysis. The innovative proposed method based on the use of DES resulted in an efficient greener alternative to the conventional one for determining phenolic compounds in rocket samples.

## 2. Results and Discussion

### 2.1. Screening of DESs: Selection of the Best Extraction Solvent

**Figure 1:**
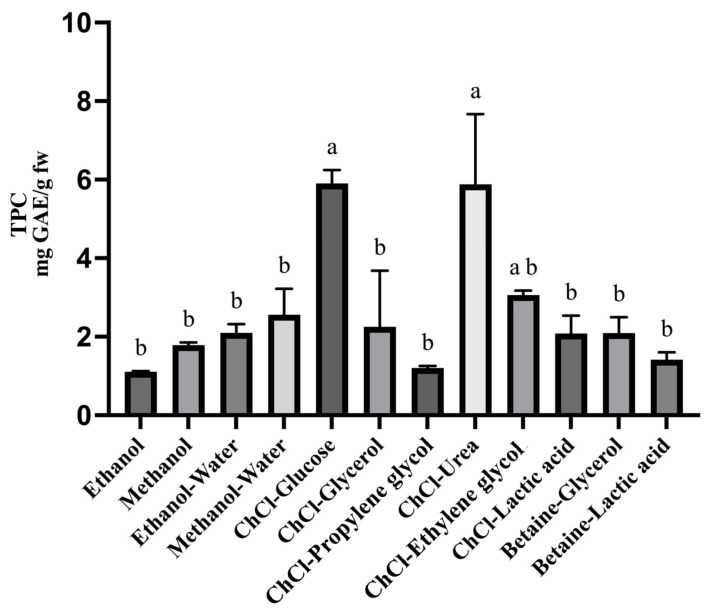
TPC of rocket extracts obtained with different DESs and traditional solvents. A one-way analysis of variance (ANOVA) followed by Tukey’s test was performed to define statistically significant differences. Different letters indicate statistically significant differences among results.

Traditional extraction methods to obtain phenolic compounds from natural sources involve the use of high amounts of organic solvents (methanol, chloroform, and hexane) [21,22]. The risk of the permanence of harmful residues in the extract limits its use, and in this context, DESs represent a very promising alternative in the field of extractions from natural sources [23]. For an effective and selective extraction via DES, an accurate optimization process is necessary. In fact, these two parameters are influenced by different factors such as type of DES, quantity of water to add to the DES in order to reduce its viscosity, time and temperature of extraction, and matrix-to-solvent ratio. The first step of the method optimization involved the selection of the best extractive solvent. Although in the literature, the most used solvents for phenolic compounds extraction from *Eruca sativa* Mill. were ethanol, acetone, and ethyl acetate [24], in this current study, it has been shown that both pure methanol and the mixture of methanol–water 80% provided a higher yield in terms of TPC (Figure 1) compared with the TPCs indicated in the literature [1,8,12,24]. All of the extraction procedures were performed as follows: an amount of 0.1 g of fresh rocket was added with 5 mL of each DES, added with 30% (*v*/*v*) of water, and put in an ultrasound bath (Elmasonic S30H, Elma Schmidbauer GmbH, Singen, Germany) at 50 °C for 45 min. No statistical differences were detected among the TPC obtained with the different tested organic solvents. Also, the efficiency of the considered DESs (ChCl-glucose; ChCl-propylen glycol; ChCl-urea; ChCl-ethylene glycol; ChCl-lactic acid; betaine-glycerol; betaine-lactic acid) were evaluated. All tested DESs guaranteed an extraction yield comparable or better than organic solvents. DESs with the best extractive yield were ChCl-glucose (1:2 molar ratio) with a TPC of 5.9 ± 0.3 mg GAE/g and ChCl-urea (1:2 molar ratio) with a TPC of 5.8 ± 1.8 mg GAE/g; however, the extraction using the ChCl-urea DES was not reproducible, probably due to its high viscosity. A DES consisting of ChCl and glucose, which is considered a NADES, only composed by natural products, was selected for the successive optimization phase.

### 2.2. FTIR Characterization

**Figure 2:**
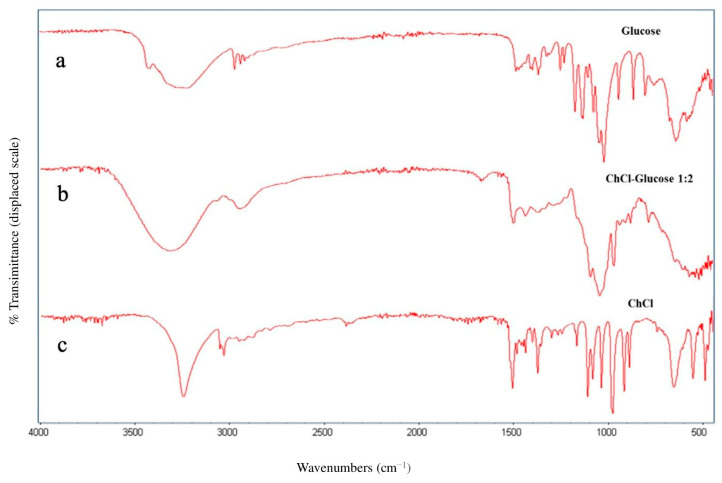
FTIR spectra of glucose (**a**), ChCl-glucose DES (**b**) and ChCl (**c**), respectively.

A ChCl-glucose DES characterization study was performed using Fourier transform infrared spectroscopy (FTIR) in the attenuated total reflectance (ATR) mode. An ATR analysis confirmed the structure of the DES. The frequencies of the bonds present in the DES (b) were compared with those of the corresponding individual components, ChCl (c) and glucose (a), respectively (Figure 2). The band at 3210 cm^−1^ in the ChCl spectrum is associated with stretching of the -OH bond; the bands between 2800 and 3000 cm^−1^ are linked to the CH_3_ and CH_2_ stretching, while the C–H scissor and bending are at 1450–1290 cm^−1^. In the fingerprint region, the stretching of alcoholic CO at 1270 cm^−1^, COC and COH bending, and ammonium CN stretching in the range of 1100–950 cm^−1^ are visible [21]. The characteristic peaks for betaine are observed at around 1400 cm^−1^ (CN stretching) and 1323 cm^−1^ (COO stretching), while the peak at 1625 cm^−1^ corresponds to the asymmetric stretching of the carboxylate group.

### 2.3. Optimization of the Water Percentage to Add to the DES

**Figure 3:**
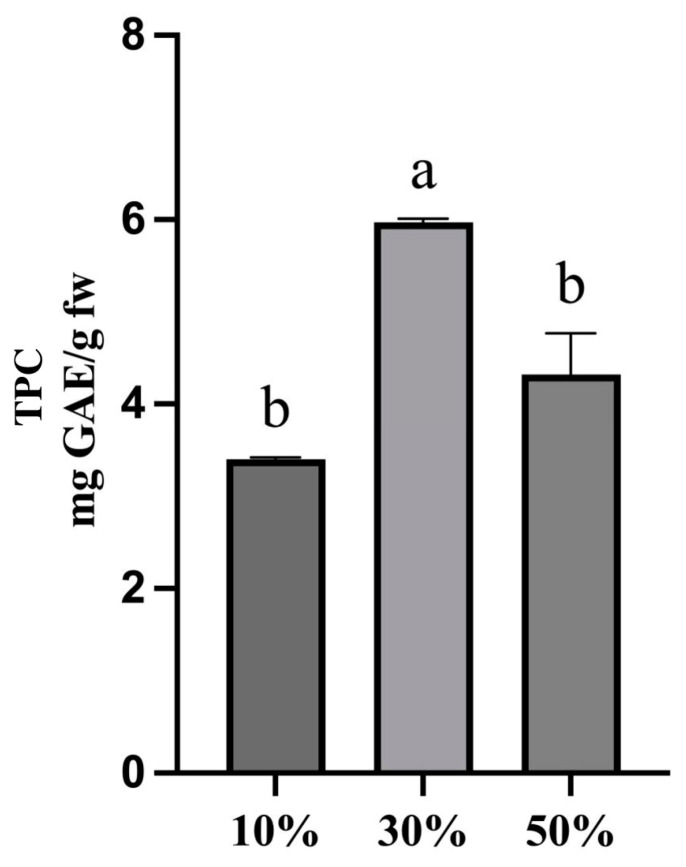
Concentration (mg GAE/g) of phenolic compounds extracted from rocket varying the percentage of water added to the selected DES. A one-way analysis of variance (ANOVA) followed by Tukey’s test was performed to define statistically significant differences. Different letters indicate statistically significant differences among results.

The presence of water is one of the aspects that most influences the application of eutectic solvents in both positive and negative modes, as excessive additions completely dissolve DESs hydrogen bonds, while a high quantity of water decreases DESs’ viscosities. Furthermore, the addition of water to the DES can modulate DES polarity [25]. Once the ChCl-glucose (1:2 molar ratio) DES was selected as an extraction solvent, the amount of water to add to it was optimized. The extraction was carried out as follows: a volume of 5 mL of ChCl-glucose (1:2 molar ratio), which ensured the best extractive yield in terms of TPC during the previous optimization phase, were added to 0.1 g of rocket. Different volumes of water were added to the selected DES: 10% (*v*/*v*), 30% (*v*/*v*), and 50% (*v*/*v*), and the extraction was performed with an extraction time of 45 min and an extraction temperature of 50 °C. As shown in Figure 3, the best extraction yield was obtained by adding 30% (*v*/*v*) of water to the DES, probably because the addition of 50% water led to a decrease in yield because, as already explained before, excessive additions of water completely dissolve the hydrogen bonds, breaking the DES structure [25]. A quantity of water of 30% (*v*/*v*) of the volume DES was selected as the optimal condition for the optimized extraction.

### 2.4. Optimization of the Matrix-to-Solvent Ratio

**Figure 4:**
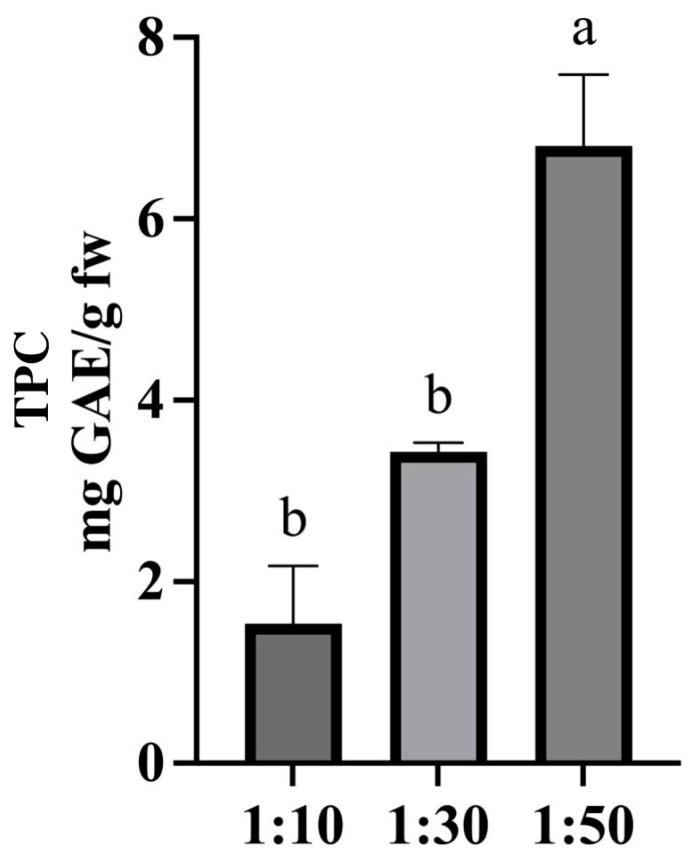
Concentration (mg GAE/g) of phenolic compounds obtained considering different quantities of the matrix-to-solvent ratio. A one-way analysis of variance (ANOVA) followed by Tukey’s test was performed to define statistically significant differences. Different letters indicate statistically significant differences among results.

Among the main parameters influencing extractive efficiency, the matrix-to-solvent ratio plays a crucial role on the concentration of phenolic compounds [26]. Using ChCl-glucose (1:2 molar ratio) DES added with 30% water, previous extraction procedures were carried out by testing different matrix-to-solvent ratios to identify which ratio guaranteed a better extractive yield. As shown in Figure 4, as the matrix-to-solvent ratio increased, the determined TPC also increased, passing from 1.54 ± 0.6 mg GAE/g with a ratio of 1:10 (*w*/*v*) to 6.8 ± 0.7 mg GAE/g with a ratio of 1:50 (*w*/*v*). A matrix-to-solvent ratio of 1:50 (*w*/*v*) was further selected as the optimal extraction condition.

### 2.5. Optimization of the Time and Temperature of Extraction

**Figure 5:**
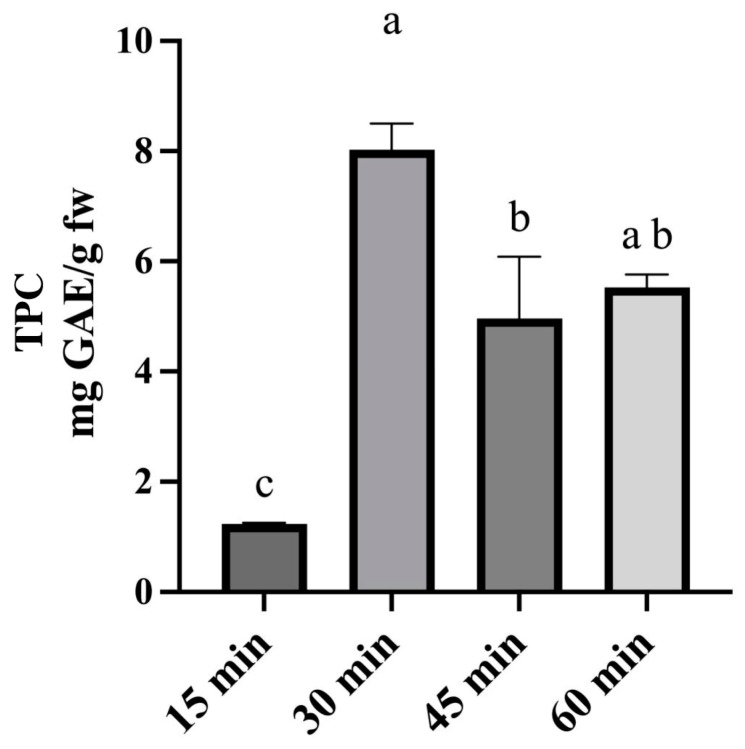
Concentration (mg GAE/g) of phenolic compounds obtained by testing different extraction times. A one-way analysis of variance (ANOVA) followed by Tukey’s test was performed to define statistically significant differences. Different letters indicate statistically significant differences among results.

Because the best matrix-to-solvent ratio was selected, extraction time and extraction temperature were optimized. Firstly, the optimization of the extraction procedure involved the selection of the extraction time that guaranteed the best result in terms of obtained TPC. The extraction procedures were performed as follows, considering four different extraction times (15 min, 30 min, 45 min, and 60 min): an amount of 0.1 g of fresh rocket were added with 5 mL of ChCl-glucose (1:2) and put in an ultrasound bath (Elmasonic S30H, Elma Schmidbauer GmbH, Singen, Germany) for 15 min, 30 min, 45 min, and 60 min at 50 °C. As can be seen in Figure 5, an increase in the extraction time from 15 min to 30 min increased the extraction yield, while too long of an extraction time caused a phenolic compounds degradation with a low obtained TPC [27]. A time of 30 min was selected for the further optimization phase.

Performing the extraction as previously described and considering an extraction time of 30 min, the extraction temperature was optimized. Specifically, the extraction was carried out at three different extraction temperatures of 30, 50, and 80 °C.

**Figure 6:**
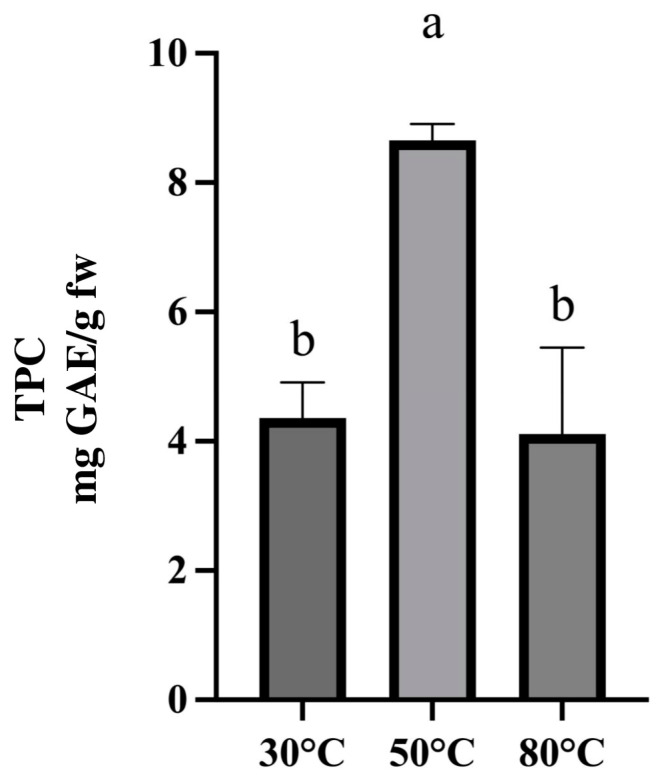
Concentration (mg GAE/g) of phenolic compounds obtained testing different extraction temperatures. A one-way analysis of variance (ANOVA) followed by Tukey’s test was performed to define statistically significant differences. Different letters indicate statistically significant differences among results.

As possible to see in Figure 6, an increase in the extraction yield was obtained by increasing the extraction temperature from 30 to 50 °C. A higher extraction temperature can favor mass transfer improving the phenolic compounds extraction yield. However, performing the extraction with an extraction temperature set at 80 °C caused a phenolic compounds degradation [27]. The optimized extraction procedure involved weighing 0.1 g of rocket in a tube, adding 5 mL of ChCl-glucose (1:2) DES with 30% *v*/*v* of water, and putting the mixture into an ultrasound bath (Elmasonic S30H, Elma Schmidbauer GmbH, Singen, Germany) for 30 min at 50 °C.

### 2.6. Comparison of the Presented Method with Those Reported in the Literature

The optimized extraction procedure was guaranteed to obtain a TPC 8.45 ± 0.11 mg GAE/g fw under the following extraction conditions: ChCl-glucose (1:2), added with 30% (*v*/*v*) of water, was used as an extraction solvent, considering a quantity of the matrix to volume of solvent ratio of 1:50 *w*/*v* and performing extraction for 30 min at 50 °C. Table 1 reports the extraction conditions and TPC of other SLE procedures developed for phenolic compounds determination in different rocket samples. Only a few works about phenolic compounds determination in rocket are reported in the literature; for this reason, Table 1 shows all of the work about SLE applied to rocket samples to determine phenolic compounds. Heimler et al. [11] developed an extraction procedure for phenolic compounds from some species of freshly consumed salads, comprising ten different genotypes including Eruca sativa, using an ethanol–water mixture acidified with formic acid as the extraction solvent. Although the TPC obtained was comparable with that of our proposed method, the extraction solvent was less green than the ChCl-glucose selected DES, and the extraction time was much longer in comparison with the 30 min extraction time of this work. The extraction of phenolic compounds from rocket with ethanol and formic acid is a very effective choice to extract phenolic compounds, especially for polar compounds; furthermore, ethanol is considered a “green” solvent compared with traditional organic solvents such as methanol or acetone, and for this reason, it is safe for use in food and pharmaceutical applications as well as having a reduced environmental impact. However, formic acid, at high concentrations, can degrade some thermolabile compounds present in plant matrices and lead to the degradation of some bioactive molecules; meanwhile, in this work, the use of DES for extraction ensures extraction without degrading the phenolic compounds. All works reported in Table 1 involved a quantity of matrix/volume to solvent ratio lower than that of our proposed method but used an organic solvent as the extraction medium. Only Matev et al. [8] developed an extraction procedure characterized by the same quantity of matrix/volume to solvent ratio of the present work but used an extraction solvent composed of hexane and petroleum ether, and they achieved a lower extraction yield. It is important to specify that in this work Matev et al. [8] did not use fresh leaves but dried and ground plant material. However, the environmental and health risks, together with the limited selectivity for more polar compounds such as phenols, make this type of solvents progressively replaced by more sustainable alternatives in the extraction processes. The extraction of phenolic compounds with the methanol–water mixture 50:50 *v*/*v* performed by Romano et al. [1] and Toledo-Martín et al. [2] is a common technique that exploits the high polarity of the mixture to extract a wide range of phenolic compounds. In the study of Romano et al. [1], samples belonging to different species of rocket (*Diplotaxis tenuifolia* L.) have been analyzed, and the result is expressed in dry weight. Likewise, in the study conducted by Toledo Martin et al. [2], the phenolic content of the leaves of rocket belonging to the species (*Eruca vesicaria*) was evaluated and expressed in terms of dry weight. Compared with the DES used in this study, the extraction technique performed by Toledo-Martín et al. [2] also presents obvious disadvantages from an environmental and sustainability point of view because, although the water–methanol mixture is relatively effective, the use of methanol has a negative environmental impact. In this study, DESs were used for the extraction of phenolic compounds from *Eruca sativa* Mill., which have a low environmental impact, lower toxicity, and greater sustainability. Due to their strong hydrogen bonding ability, DESs can dissolve a wide range of phenolic compounds better than many traditional solvents, enhancing extraction efficiency. The use of DES as an extraction solvent allowed us to obtain a high value of TPC performing a rapid and safe extraction procedure, reducing environmental impact.

### 2.7. Green Analytical Procedure Index (GAPI) Analysis

**Figure 7:**
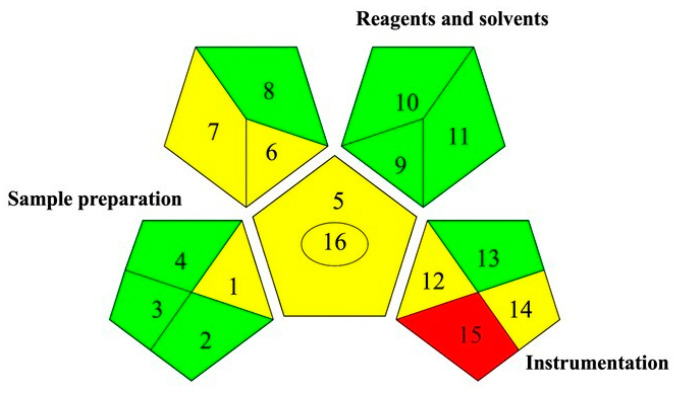
Complex GAPI analysis. Collection (1); preservation (2); transport (3); storage (4); type of method (5); scale of extraction (6); solvents/reagents used (7); additional treatments (8); amount (9); health hazard (10); safety hazard (11); energy (12); occupational hazard (13); waste (14); waste treatment (15); type of analysis (16).

To evaluate the actual greenness of the method, Complex GAPI v.0.2 was used. It is a software used for the selection of analytical procedures less harmful to the environment, thus allowing us to compare the environmental impact of different methods and reduce it to a minimum. The software returns five pentagrams that present a different color ranging between yellow, green, and red depending on each phase of the analytical method (e.g., sample transport, health risks, and instrumental energy expenditure). The five pentagrams instead represent the preparation of the sample, the reagents and the solvent used, the instrumentation, and the qualitative or quantitative purpose of the analytical method. As can be observed in Figure 7, the central pentagram is yellow as a quantitative analysis of the obtained extract was performed through spectrophotometric analysis, and the phenolic compounds profile of the extract was determined using an HPLC-MS analysis. The developed analytical method guarantees a high greenness, although the waste treatment is a parameter that needs to be improved. The pentagram related to used reagents and solvents is all filled with green color as a DES, specifically a NADES, and in a low volume, it was used as an extraction solvent to determine phenolic compounds in rocket.

### 2.8. HPLC-PDA/ESI-MS Qualitative Analysis

**Figure 8:**
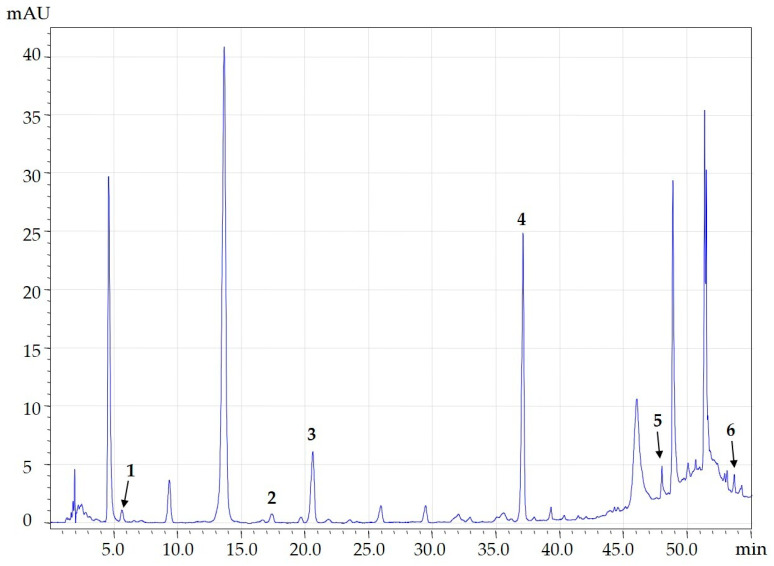
HPLC-PDA chromatogram (λ = 280 nm) of rocket extract obtained using optimized conditions and the phenolic compounds putatively identified: isorhamnetin (1); quercitin-3-O-galactoside (2); roseoside (3); quercitin (4); ferulic acid (5); chlorogenic acid (6).

Rocket has been increasingly studied for the presence of bioactive molecules with antioxidant and anti-inflammatory properties that are deeply exploited both in the field of cosmeceutical and nutraceutical industries. The main phenolic compounds found in *Eruca sativa* Mill. are: flavonols (kaempferol-3-O-rutinoside, quercetin), cinnamic acids (p-coumaric, ferulic and chlorogenic acids, and benzoic acid derivatives (protocatechuic) [28]. To identify phenolic compounds in *Eruca sativa* Mill., the extracts obtained with the optimized extraction method, described in Section 3.5, were analyzed through an HPLC-PDA/ESI-MS analysis. The identification of phenolic compounds was carried out considering both retention time (t_R_), UV spectra data, MS spectra, use of standard compounds and the data available in the literature. In total, six phenolic compounds were detected and identified considering their *m*/*z* [M-H]^−^ and comparing the retention times with those of the available standard molecules: isorhamnetin (*m*/*z* = 316); quercitin-3-O-galactoside (*m*/*z* = 463); roseoside (*m*/*z* = 385); quercitin (*m*/*z* = 301); ferulic acid (*m*/*z* = 193); and chlorogenic acid (*m*/*z* = 353) (Figure 8).

### 2.9. Treated Samples Analysis

The optimized extraction procedure was used to determine phenolic compounds in rocket treated samples growth under controlled induced conditions of stress. According to the literature, this kind of stress is supposed to increase bioactive molecules accumulation in plants [28,29]. Rocket samples were analyzed under the optimized following conditions, involving weighing 0.1 g of rocket in a tube, adding 5 mL of ChCl-glucose (1:2 molar ratio) DES with 30% *v*/*v* of water, and putting the mixture in an ultrasound bath (Elmasonic S30H, Elma Schmidbauer GmbH, Singen, Germany) for 30 min at 50 °C. No differences were revealed in the TPC determined in the three rocket samples treated without and with 150mM and 300 mM NaCl, being 8.5 ± 1.7 mg GAE/g fw, 7.1 ± 0.9 GAE/g fw and 8.2 ± 0.6 GAE/g fw, respectively. The salt concentrations used were probably not sufficient for inducing an increase in the accumulation of phenolic compounds.

## 3. Materials and Methods

### 3.1. Chemicals

The chemicals used in this work include solvents used for the HPLC-MS analysis and extraction procedure, such as methanol (MeOH) (99.9%), ethanol (EtOH) (99.9%), water (HPLC-MS grade), and formic acid (95–97%), all supplied by Sigma-Aldrich (Milan, Italy). The chemicals used for the Folin–Ciocalteu test, including gallic acid (97.5%), sodium carbonate (>99.5%), and Folin–Ciocalteu reagent, were also supplied by Sigma-Aldrich (Milan, Italy). Both the macro- (potassium nitrate, calcium nitrate, magnesium sulfate, and ammonium phosphate) and microelements (ethylenediaminetetraacetic acid, ferric-sodium salt (phena-2,2′,2″,2‴-(Ethane-1,2-diyldinitrilo)tetraacetic acid, and sodium silicate) and micronutrients (copper sulfate, boric acid, zinc sulfate, and manganous chloride) used for the hydroponic solution were provided by Sigma-Aldrich (Milan, Italy). Finally, for the preparation of DESs, betaine, ChCl, lactic acid, urea, glycerol, ethylene glycol, and propylene glycol were provided by Carlo Erba (Milan, Italy).

### 3.2. Samples

The *Eruca sativa* Mill. used for method optimization was purchased from a local market and minced prior to being subjected to the extraction procedure. Specifically, the commercial rocket was grown in Piana del Sele IGP in the province of Salerno. The farms were selected in the immediate vicinity of the Battipaglia (SA) plant.

Treated *Eruca sativa* Mill. samples, analyzed with the optimized method, consisted of plants growth by seeds of *Eruca sativa* Mill. purchased from ZenGreens^®^ (Leverkusen, Germany) and cultivated as reported in Section 3.10.

### 3.3. DESs Preparation

For DESs preparation, quantities of HBA and HBD were weighed according to specific molar ratios and placed in a tube. Subsequently, the tube containing the mixture of the two components was placed in the bath (Elmasonic S30H, Elma Schmidbauer GmbH, Singen, Germany) at a temperature of 80 °C until a homogeneous and transparent solution was formed. To prove the effective formation of the eutectic mixtures, the infrared spectra of the single HBA and HBD and the formed DES were acquired.

### 3.4. FTIR DES Characterization

The infrared spectra of the single HBA and HBD and the formed DES were acquired to establish the effective formation of the eutectic mixture. All of the analyses were conducted at room temperature with an Agilent Cary 630 FTIR spectrometer (Agilent Technologies, Santa Clara, CA, USA) equipped with MicroLab Expert FTIR v.1.3 0.0 software. The spectra were acquired in air at room temperature using a diamond attenuated total reflectance (ATR) cell, with six replicates over the range from 400 to 4000 cm^−1^ (64 scans for acquisition, 4 cm^−1^ resolution).

### 3.5. Extraction Method

The extraction procedure was relatively simple and, unlike other works present in the literature [11], did not require the use of liquid nitrogen. The extraction of phenolic compounds was carried out according to the following procedure: firstly, 0.1 g of shredded *Eruca sativa* Mill. were weighed in a tube and then cut into very small pieces. Subsequently, 5 mL of DES was added to the shredded rocket and then stirred for about 30 s with vortexing. The samples were placed in an ultrasonic bath (Elmasonic S30H, Elma Schmidbauer GmbH, Singen, Germany) for 30 min at 50 °C and then centrifuged for 3 min at room temperature at a rate of 5000 rpm. The supernatant was then recovered and diluted 1:10 (*v*/*v*) with water before performing the spectrophotometric analysis.

### 3.6. Extraction Procedure Optimization

The optimization of the extraction procedure firstly involved the study of the best DES among eight selected DESs, composed by ChCl and betaine as the HBA, while glycerol, lactic acid, urea, ethylene glycol, propylene glycol, and glucose composed the HBD (Table 2). All of the extraction procedures were performed as follows: an amount of 0.1 g of fresh rocket was added with 5 mL of extraction solvent, with each DES being added with 30% (*v*/*v*) of water and put in an ultrasound bath (Elmasonic S30H, Elma Schmidbauer GmbH, Singen, Germany) at 50 °C for 45 min.

Once the most efficient DES was selected, some parameters were optimized: quantity of water to add to DES with the aim of reducing its viscosity, matrix-to-solvent ratio, and time and temperature of the extraction. For each parameter optimization, TPC was selected as response and extraction procedures were carried out as previously described, and we set the last optimized parameter at the selected value. The quantity of water to add to the selected DES was evaluated in a range from 10% to 50%. According to the literature, a water percentage higher than 50% could cause the break of the DES with the decrease in the extraction yield [21]. Matrix-to-solvent ratios of 1:10, 1:30, and 1:50 (*w*/*v*) were tested to select the one that guarantees recovering the highest total phenolic compounds concentration. Four different extraction times ranging between 15 and 60 min (15 min, 30 min, 45 min, 60 min) and three different extraction temperatures ranging between 30 °C and 80 °C (30 °C, 50 °C, 80 °C) were evaluated to select the best extraction conditions. All these parameters were considered to identify those that ensured a higher extraction yield of phenolic compounds from rocket. For each optimization step, three replicates of the tested extraction procedures were performed, and a one-way analysis of variance (ANOVA) followed by Tukey’s test, through GraphPad Prism v.8.0.1 software, was performed to define statistically significant differences (*p* < 0.05).

### 3.7. Determination of Total Phenolic Content (TPC)

First, 20 μL of 1:10 *v*/*v* diluted sample was added to 1580 μL of distilled water and 100 μL of Folin–Ciocalteu reagent and stored in the dark for 8 min. Then, 300 μL of an aqueous solution of Na_2_CO_3_ 0.2 g/mL was added into the tube, and the mixture was maintained in the dark for 2 h under continuous stirring. After this, 200 μL of each sample was taken and tripled into the 96-well Greiner plate. To perform spectrophotometrical analysis, a blank with the diluted 1:10 *v*/*v* extraction solvent was used for each DES. The absorbance was measured with an Infinite M200 PRO Tecan microplate spectrophotometer (Tecan Trading AG, Männedorf, Switzerland) at 765 nm. The calibration curve was prepared using gallic acid as the standard in a range from 0 to 2000 µg/mL. Phenolic compounds concentrations were expressed as milligrams of gallic acid equivalent (mg GAE) per 100 g of fresh weight rocket.

### 3.8. HPLC/ESI-MS Qualitative Analysis

The extract characterization was performed using a Shimadzu Prominence LC-20A chromatograph (Shimadzu, Milan, Italy) equipped with two LC-20 AD XR pumps-a SIL-10ADvp pumps (Shimadzu, Milan, Italy), the CTO-20 AC-column furnace, and a DGU-20 A3 degasser coupled with a PDA SPD-detectorM10Avp PDA and mass spectrometer (LCMS-2010, Shimadzu, Tokyo, Japan) equipped with an electrospray interface (ESI). MS data were acquired using Shimadzu LC solution version 3.7 (Shimadzu, version 3.7). The optimized analytical method was performed by setting the following conditions: analytes separation was carried out using an Ascentis^®^ Column for HPLC (3 μm particle size, L × I.D. 15 cm × 4.6 mm) (Merck KGaA, Darmstadt, Germany). The elution of the analytes was carried out at a constant flow rate of 1 mL/min at a temperature of 40 °C. The mobile phase (A) consisted of water, while the mobile phase (B) was methanol acidified with 0.1% (*v*/*v*) of formic acid. The gradient elution used for the separation of bioactive compounds was: t = 0’ 10%B; t = 30’ 30%B t = 40’ 45%B; and t = 50’ 100%B with an injection volume of 2 μL. Mass acquisition (MS) was performed in single ion monitoring (SIM) and FULL SCAN in the negative mode, setting the following parameters: nebulizing gas flow (N_2_): 1.5 mL/min; event time: 1 sec; scanning speed: 1000 Amu/sec; detector voltage: 1.5 kV; interface temperature: 250 °C; CDL temperature: 300 °C; thermal block: 300 °C; RF: 150.0 V.

### 3.9. GAPI Index Analysis

ComplexGAPI v.0.2 software was used to define the GAPI pictogram. The GAPI metric describes the greenness of each step of the analytical process, from the collection of samples until the final analysis. The GAPI pictogram is composed by 5 pentagrams, which represent the sample preparation, the reagents and solvent used, the instrumentation, and the qualitative or quantitative purpose of the analytical method. Each pentagram takes a color on the base of its greenness: green color is associated with a sustainable step of the analytical method, while medium and high environmental impact step have yellow and red colors, respectively.

### 3.10. Real Sample Growth Conditions

The seeds of *Eruca sativa* Mill. were purchased from ZenGreens^®^. After that, the seeds were grown in the Grodan rock wool previously wetted under cold water, and then, the seeds were put in cultivation for a month. During this time, the seeds were watered three times a week, with hydroponics consisting of: macroelements (potassium nitrate, calcium nitrate, magnesium sulfate, and ammonoium phosphate); microelements such as ethylenediaminetetraacetic acid, ferric-sodium salt (phena-EDTA) and sodium silicate; and microelements (copper sulfate, boric acid, zinc sulfate, and manganous chloride). After one month of hydroponics, three days before sampling, the rocket plants were divided into three groups and treated in hydroponics, hydroponics with NaCl 150 mM, and hydroponics with 300 NaCl mM, respectively. Finally, on the day of sampling, the plants were collected and analyzed.

## 4. Conclusions

The current work aimed at the development of an eco-friendly extraction method for phenolic compounds determination in rocket (*Eruca sativa* Mill.) followed by a qualitative HPLC-ESI/MS analysis of the extract. To the best of our knowledge, this work represents the first attempt in which DESs replace traditional organic solvents for the extraction of polyphenols from *Eruca sativa* Mill., meeting the requirements of green chemistry. A green approach for extracting phenolic compounds from *Eruca sativa* Mill. based on the use of an eco-friendly extraction solvent was developed and optimized. The innovative green extraction method for total phenolic compounds determination in rocket resulted in a valid alternative to the conventional one, based on the use of organic solvent. In fact, the use of ChCl-glucose DES as an extractive solvent also ensured a better yield in terms of TPC in comparison with organic solvents. Also, the use of DES allowed us to pursue and respect the principles of green chemistry, which aims to use and generate substances that possess little or no toxicity to human health and the environment. Thanks to their features, including ease of preparation, low costs, stability in the presence of water, and especially their high biodegradability and very low toxicity of components, DESs represent solvents with high potential, especially for extraction from natural sources. From the application of the optimized method to the analysis of real plant samples grown under different salt stress conditions, there were no differences in the accumulation of phenolic compounds. Different salt concentrations will probably have to be evaluated. However, the developed method represents a green, innovative, and feasible strategy for the determination of phenolic compounds in rocket samples.

## Figures and Tables

**Table 1:** Comparison of the proposed method with those reported in the literature.

Sample	Solvent	Extraction Technique	Quantity of Matrix	Volume of Solvent	Extraction Time	Extraction Temperature	TPC	Refs.
*Eruca sativa*	Hexane: petroleum ether mixture (1:1, *v*/*v*)—50% ethanol	SLE	1 g	50 mL	30 min	30 °C	4.45 ± 0.14 mg GAE/g dry weight	[8]
*Eruca sativa*	Ethanol–water (70:30, *v*/*v*) pH 3.2 by formic acid)	SLE	2 g	30 mL	Overnight	Room temperature	2. 08 ± 0.18 mg GAE/g fresh weight	[11]
*Eruca sativa*	Methanol–water (70:30, *v*/*v*) pH 3.2 by formic acid)	SLE	0.1 g	2.5 mL	15 min	75 °C	2–12 mg GAE/g dry weight	[2]
*Eruca sativa*	Water/methanol (50:50, *v*/*v*)	SLE	0.5 g	20 mL	-	-	3–5 mg GAE/g dry weight	[1]
*Eruca sativa*	ChCl-glucose (1:2)	SLE	0.1 g	5 mL	30 min	50 °C	8.45 ± 0.11 mg GAE/g fresh weight	This work

**Table 2:** HBAs and HBDs and molar ratios of tested DESs.

Hydrogen Bond Donor (HBD)	Hydrogen Bond Acceptor (HBA)	Specific Molar Ratio
Lactic acid	Betaine	2:1
Glycerol	Betaine	2:1
Lactic acid	Choline chloride	2:1
Glycerol	Choline chloride	2:1
Urea	Choline chloride	2:1
Glucose	Choline chloride	2:1
Ethylene glycol	Choline chloride	2:1
Propylene glycol	Choline chloride	2:1

## Data Availability

The original contributions presented in this study are included in the article. Further inquiries can be directed to the corresponding author.

## Abbreviations

The following abbreviations are used in this manuscript:

DES	Deep eutectic solvent
ChCl	Choline chloride
HPLC	High-performance liquid chromatography
PDA	Photodiode array
ESI	Electrospray ionization
MS	Mass spectrometry
HBA	Hydrogen bond acceptor
HBD	Hydrogen bond donor
NADES	Natural deep eutectic solvent
NaCl	Sodium chloride
FTIR	Fourier transform infrared spectroscopy
SIM	Single ion monitoring
GAPI	Green analytical procedure index
EDTA	2,2′,2″,2‴-(Ethane-1,2-diyldinitrilo)tetraacetic acid
